# Marine lipopeptide Iturin A inhibits Akt mediated GSK3β and FoxO3a signaling and triggers apoptosis in breast cancer

**DOI:** 10.1038/srep10316

**Published:** 2015-05-14

**Authors:** Goutam Dey, Rashmi Bharti, Gunaseelan Dhanarajan, Subhasis Das, Kaushik Kumar Dey, B N Prashanth Kumar, Ramkrishna Sen, Mahitosh Mandal

**Affiliations:** 1 School of Medical Science and Technology, Indian Institute of Technology Kharagpur, Kharagpur-721302, West Bengal, India; 2 Department of Biotechnology, Indian Institute of Technology Kharagpur, Kharagpur-721302, West Bengal, India

## Abstract

Akt kinase is a critical component of the PI3K/Akt signaling pathway, which is frequently over expressed in human cancers including breast. Therapeutic regimens for inhibiting breast cancer with aberrant Akt activity are essential. Here, we evaluated antitumor effect of a marine bacteria derived lipopeptide ‘Iturin A’ on human breast cancer *in vitro* and *in vivo* through disrupting Akt pathway. Proliferation of MDA-MB-231 and MCF-7 breast cancer cells were significantly inhibited by Iturin A and it induced apoptosis as confirmed by increased Sub G_1_ populations, DNA fragmentation, morphological changes and western blot analysis. Furthermore, Iturin A inhibited EGF induced Akt phosphorylation (Ser473 and Thr308) and its downstream targets GSK3β and FoxO3a. Iturin A inactivated MAPK as well as Akt kinase leading to the translocation of FoxO3a to the nucleus. Gene silencing of Akt in MDA-MB-231 and MCF-7 cells reduced the sensitivity of cancer cells to Iturin A. Interestingly, overexpression of Akt with Akt plasmid in cancer cells caused highly susceptible to induce apoptosis by Iturin A treatment. In a xenograft model, Iturin A inhibited tumor growth with reduced expressions of Ki-67, CD-31, P-Akt, P-GSK3β, P-FoxO3a and P-MAPK. Collectively, these findings imply that Iturin A has potential anticancer effect on breast cancer.

The menace of chemo-resistance of the cancer cells and a steady decline in the discovery of new lead anticancer molecules has thrown a formidable research challenge to the concerned scientific community. One of the most prevalent cancers is breast cancer that is a common malignancy affecting females worldwide. It is developed due to a number of cellular and molecular transformations that lead to breast cancer cell proliferation and inhibition of apoptosis. These events involve disrupting various signaling networks and thereby resulting in altered gene expression. Among these deregulated signaling pathways, Akt/PKB plays as major contributor to the development of many cancers including breast cancer^1^,^2^. A numbers of growth factors e.g., epidermal growth factor (EGF), vascular endothelial growth factor (VEGF) and Insulin-like growth factor (IGF) activate the receptors tyrosine kinase leading to phosphorylation in kinase domain. Activated growth factor receptors ultimately induce conversion of phosphatidylinositol 4,5-bisphosphate (PIP2) to phosphatidylinositol 3,4,5 trisphosphate (PIP3) in presence of lipid kinase phosphoinositide 3-kinase (PI3K). Phosphatase and tensin homolog (PTEN) negatively regulates this conversion of PIP2 to PIP3 by phosphatase activity^3^. Akt remains in cytoplasm in an inactive form, but activated Akt translocates to membrane and binds to PIP3. This binding of Akt with membrane lipid PIP3 in pleckstrin homology (PH) domain of Akt causes phosphorylation at Thr308 on its activation loop by membrane localized 3-phosphoinositide-dependent protein kinase 1(PDK1). Phosphorylation at Ser473 is required for further activation of Akt^4^. Constitutive activation of Akt, loss of tumor suppressor PTEN, over expression of various growth factor receptors and mutation in PI3K ultimately lead to amplification of Akt signaling pathway^5^,^6^,^7^. Amplified Akt exerts its oncogenic action via triggering multiple downstream proteins^8^. These downstream proteins include Forkhead family of transcription factor (FoxO3a) and glycogen synthase kinase 3 (GSK3β). Akt directly regulates the functions of FoxO3a through phosphorylation, leading to its accumulation in the cytoplasm. Inhibition of Akt causes dephosphorylation and nuclear localization of FoxO3a, resulting in its activation. Activated FoxO3a triggers apoptosis or cell cycle arrest through down regulation of anti apoptotic proteins (Bcl-2, Bcl-xL and Mcl-1) via Bim activation^9^. Another substrate GSK3β (Ser9) induces cell apoptosis via multiple mechanisms^10^. Altered Akt signaling is well associated with biological events for e.g., tumor cell survival and proliferation, inhibition of apoptosis through up regulating Bcl-2 family proteins like Bcl-2, Bcl-xL and Mcl-1^11^,^12^. Changes of BAX conformation as well as translocation of BAX to mitochondria are inhibited by Akt leading to suppression of mitochondrial membrane potential change, Cytochrome C release, activation of caspase and apoptosis^13^. So, inhibition of Akt kinase is an attractive target for development of new anticancer molecules for breast cancer therapy.

Lipopeptides that are primary metabolites biosynthesized by a number of micro-organisms qualify as potential new generation anticancer agents against breast cancer, because of their low toxicity, easy biodegradability and their ability to kill the cancer cells selectively through various mechanisms of inhibition of signaling pathways^14^. Though there are some reports on anticancer activity of surfactin, a well known lipopeptide, against breast cancer cell lines^15^, its hemolytic property and relatively higher toxicity are the major bottlenecks^16^ in the realization of its potential as an anticancer drug. Thus, in the present study, we are reporting the anticancer activity of another lipopeptide molecule, Iturin A, purified by us from *Bacillus megaterium*, which is a marine bacterium isolated from the water sample collected from the Andaman and Nicobar Islands, India. Although many pharmacological actions of Iturin A have been reported, its anticancer property is not yet published in public domain. Our research findings indicated that Iturin A induced antiproliferative and apoptotic effect in breast cancer cells *in vitro* and *in vivo.* This apoptotic effect may be attributed to the inhibition of Akt kinase and its downstream targets FoxO3a and GSK3β.

## Results

### Purification and characterization of lipopeptide

**Figure 1:**
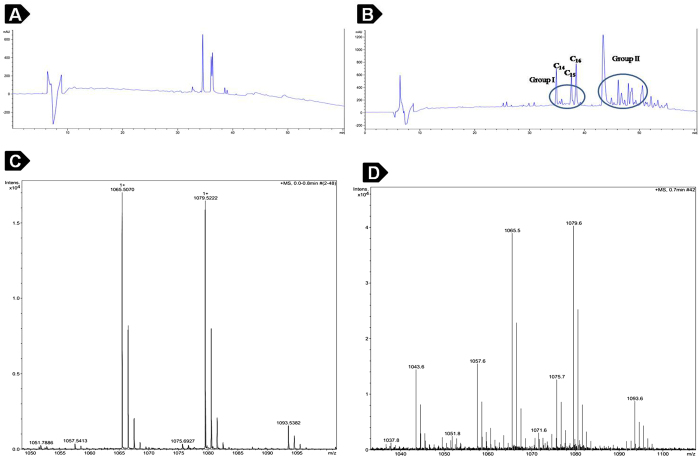
Reversed-phase HPLC chromatograms and ESI-MS analysis of the lipopeptides. (**A**) Standard Iturin A procured from Sigma. (**B**) Crude methanolic extract from the acid-precipitated supernatant fluid of *Bacillus megaterium*. HPLC conditions: Column, Zorbax C_18_; Mobile phase, milli-Q water and acetonitrile with 0.1% TFA; Flow rate, 0.4 ml min^−1^; Detection, 210 nm. (**C**) ESI-MS analysis of standard Iturin A procured from Sigma. (**D**) ESI-MS analysis of Group I lipopeptide cluster purified by reversed-phase HPLC. ESI-MS conditions: capillary voltage, 35 V; spray voltage, 4.5 kV; capillary temperature, 300 °C.

**Figure 2:**
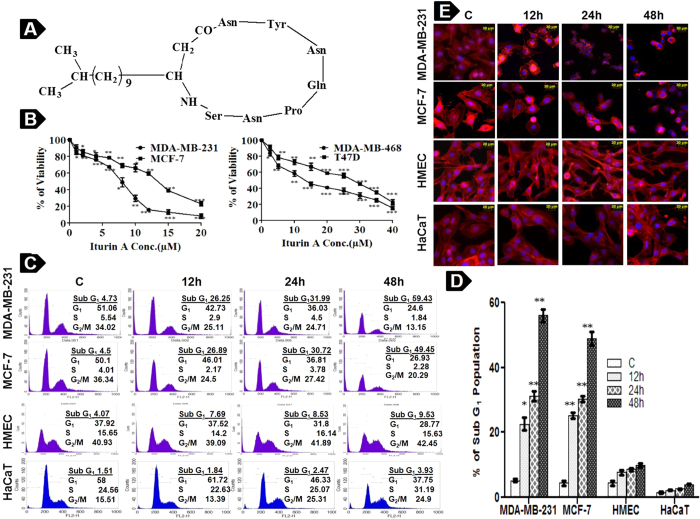
Iturin A inhibits proliferation of breast cancer cells. (**A**) Chemical structure of Iturin A consists of peptide chain containing seven amino acids linked to a fatty acid component. (**B**) MDA-MB-231, MCF-7, MDA-MB-468 and T47D breast cancer cells are treated with different concentration of Iturin A for 48 h. Following drug exposure, MTT solution is added and cells are incubated for 4 h. 100 μl DMSO was added after discarding MTT solution. Dissolved formazan is determined spectrophotometrically to measure the cell viability in treated groups compared to control. (**C**) Breast cancer cells are treated with IC_50_ dose of Iturin A and normal cells are treated with higher dose of IC_50_ dose of breast cancer cells. Representative histograms of MDA-MB-231, MCF-7, HMEC and HaCaT cells and their cell-cycle distribution after 12, 24 and 48 h of treatment are shown. (**D**) Graphical representation of Sub G_1_ populations (% of Apoptotic cells) in different cell lines from flow cytometric analysis. (**E**) Fluorescence microscopic analysis of treated MDA-MB-231 and MCF-7 cancer cells as well as HMEC and HaCaT normal cells (20 × magnification). Values are mean ± S.D of three independent experiments. Significance level is represented by *P<0.05, **P<0.01, ***P<0.001.

Iturin A present in the culture broth was collected and extracted with methanol. The methanolic extracts were subjected to further HPLC analysis. Generally, microorganisms produce lipopeptides as isoforms, which differ in the fatty acid chain length and peptide part. In the current work, HPLC chromatogram of methanol extract showed the presence of two distinct groups of isoforms. The isoforms of standard Iturin A were eluted between 34–40 min (Fig. 1A). The elution time of standard Iturin A matched with that of first group of isoforms from the methanol extract, which indicated that this group consisted of Iturin A family of lipopeptides (Fig. 1A). This was further corroborated by ESI mass spectrometry analysis of the collected fractions. The mass spectral analysis of HPLC fractions showed the presence of molecules in the range of m/z 1043–1093 Da (Fig. 1D). These peaks differ by 14 Da, indicating a series of isoforms with different fatty acid chain length. C_14_–C_16_ Iturin A isoforms were detected as both H^+^ (1043–1071) and Na^+^ (1065–1093) adducts, respectively. The mass spectra were found to be identical with that of the commercially available Iturin A (Fig. 1C). As evident from ESI-MS analysis, this group belonged to Iturin A homologues. The other group was identified as fengycin class of lipopeptides. However, C_16_ Iturin A fraction was used for further studies (Fig. 1B). Structurally, Iturin A consists of a hydrophilic peptide moiety containing amino acids with conserved sequence (Asn-Tyr-Asn-Gln-Pro-Asn-Ser) linked to a hydrophobic fatty acid chain^17^ (Fig. 2A). This lipopeptide is synthesized non ribosomally in various *Bacillus* species^18^.

### Iturin A retards cell proliferation and promotes apoptosis in breast cancer cells

**Figure 3:**
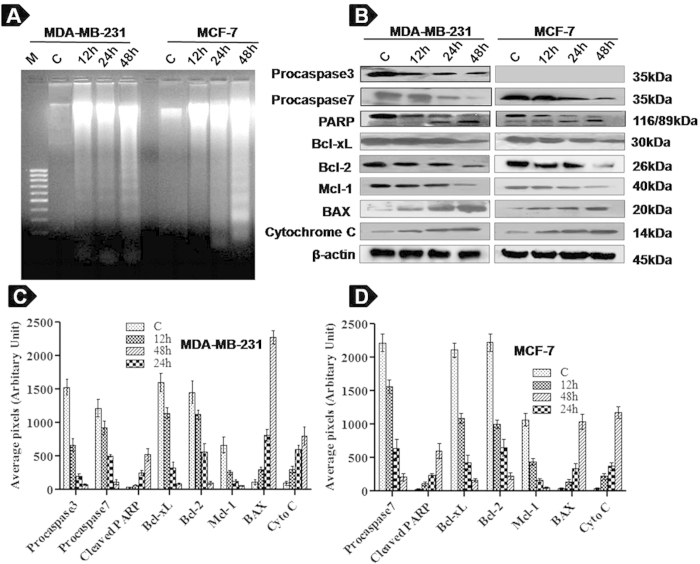
Iturin A induces apoptosis as measured by DNA laddering and western blot analysis. (**A**) DNA fragmentation by agarose gel electrophoresis in MDA-MB-231 and MCF-7 cells treated with Iturin A. (**B**) Western blot analysis of proapoptotic and anti-apoptotic proteins in MDA-MB-231 and MCF-7 cells treated with Iturin A for 12, 24 and 48 h. Blots were cut and cropped according to the molecular weight of the proteins. (**C**) and (**D**) Densitometric plot of proapoptotic and antiapoptotic proteins of MDA-MB-231 and MCF-7 cells treated with Iturin A. Each bar represents average arbitrary pixel of three experiments.

The anti-proliferative action on breast cancer cells was determined by MTT assay. MDA-MB-231 and MCF-7 cells were treated with 0–20 μM of Iturin A as well as T47D and MDA-MB-468 cells were treated with 0–40 μM of Iturin A for 48 h. The IC_50_ (50% inhibitory concentration) values of MDA-MB-231, MCF-7, MDA-MB-468 and T47D cells were 7.98 ± 0.19, 12.16 ± 0.24, 13.30 ± 0.97 and 26.29 ± 0.78 μM respectively (Fig. 2B). MDA-MB-231 and MCF-7 cells were selected for further study as they were highly sensitive to Iturin A. Next, we performed flow cytometry, cell cytoskeleton analysis and DNA ladder assay to check apoptotic effect of Iturin A in breast cancer cells. Cell cycle analysis revealed time dependent increment of Sub G_1_ population in treated MDA-MB-231 and MCF-7 cells (Fig. 2C, D). But, no significant changes of Sub G_1_ population were observed in normal HMEC and HaCaT cells (Fig. 2C, D). Cytoskeleton remodeling was observed in Iturin A treated MDA-MB-231 and MCF-7 cells (Fig. 2E). Interestingly, no significant morphological changes occurred in HMEC and HaCaT cells (Fig. 2E). Noticeable DNA ladders were observed in Iturin A treated MDA-MB-231 and MCF-7 cells (Fig. 3A).

### Iturin A alters expression profile of proteins involved in apoptosis

Apoptosis is tuned by a fine balance between survival proteins and apoptotic proteins. According to our study, Iturin A caused significant changes in expresion level of various proteins involed in apoptosis (Fig. 3B, C, D). Sequential activation of caspase plays a critical role in final steps of apoptosis. Iturin A treated groups showed prominent decrease of Procaspase3 expression in MDA-MB-231 cells in a time depemdent manner. Procaspase7 expression was also reduced in Iturin A treated MDA-MB-231 and MCF-7 cells indicating the involvement of caspase in Iturin A induced apoptosis. Cleavage of PARP (Poly ADP ribose polymerase) and release of Cytochrome C were also observed in treated groups. Further, proapoptotic BAX was upregulated as well as anti-apoptotic proteins Bcl-2, Bcl-xL and Mcl-1 were downregulated in MDA-MB-231 and MCF-7 cells treated with Iturin A in time dependent manner.

### Iturin A interferes Akt Phosphorylation and its downstream targets

**Figure 4:**
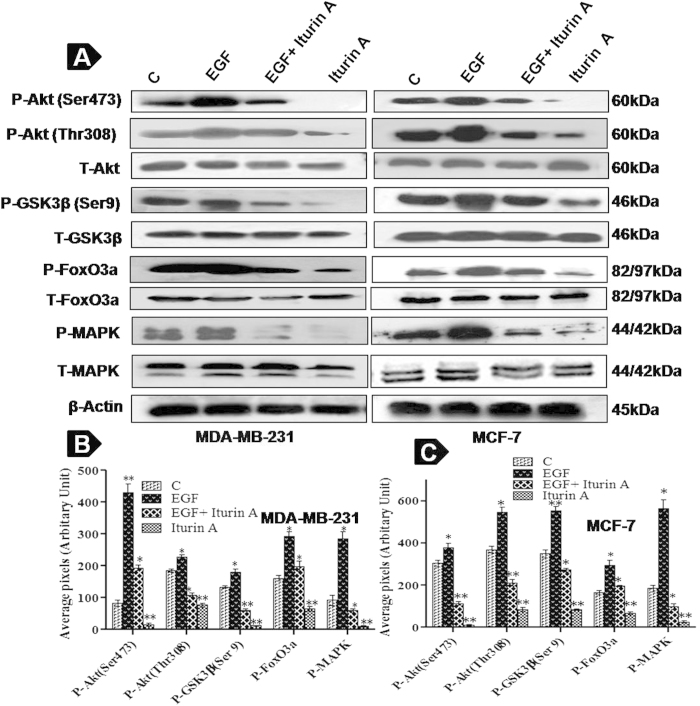
Iturin A inhibits Akt signaling pathway. (**A**) Phosphorylation levels of Akt (Ser473 and Thr308), GSK3β, FoxO3a and MAPK (p44/42) are estimated along with corresponding total proteins expression in cells grown in serum-free medium and cells stimulated with recombinant human EGF (25 ng/ml) for 30 min alone and in the presence of Iturin A. Blots were cut and cropped according to the molecular weight of the proteins. (**B**) Graphical representation of phosphorylation level of Akt, GSK3β, FoxO3a and MAPK in treated MDA-MB-231 cells. (**C**) Graphical representation of phosphorylation level of Akt, GSK3β, FoxO3a and MAPK in treated MCF-7 cells. Data are represented as mean ± S.D of three independent experiments. Significance level is represented by *P<0.05, **P<0.01, ***P<0.001.

Phospho specific western blot analysis indicated that Iturin A caused sharp down regulation of EGF induced phosphorylation of Akt (Ser473 and Thr308) and its downstream proteins GSK3β and FoxO3a in treated groups without altering total proteins in both cell lines (Fig. 4A, B, C). We also observed that Iturin A inhibited basal level (Control) as well as EGF induced expression of all phospho proteins in both cells. Moreover significant inhibition of P-MAPK was also observed in Iturin A treated MDA-MB-231 and MCF-7 cells (Fig. 4).

### Modulation of Akt activity with transfections influences sensitivity of breast cancer cells to Iturin A

**Figure 5:**
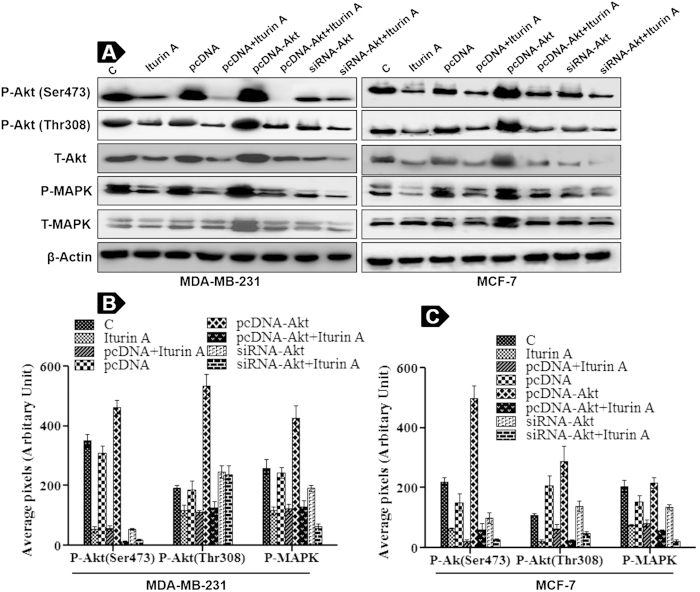
Transfection study. (**A**) MDA-MB-231 and MCF-7 cells are transfected with pcDNA, pcDNA-Akt and siRNA-Akt following Iturin A treatment. Expression levels of P-Akt (Ser 473 and Thr 308) and P-MAPK are evaluated by immunoblotting. β-Actin is used as loading control. Blots were cut and cropped according to the molecular weight of the proteins. (**B**) and (**C**) Densitometric plot of P-Akt (Ser 473 and Thr 308) and P-MAPK levels in both cell lines. Each bar represents mean ± S.D from three different experiments.

To verify the involvement of Akt kinase in Iturin A induced cell death, pcDNA-Akt plasmid and signal silence® siRNA-Akt were stably transfected in MDA-MB-231 and MCF-7 cells. Western blot analysis demonstrated increase phosphorylation status of Akt in pcDNA-Akt transfected groups compare to controls and pcDNA groups. In contrast, phosphorylation status of Akt was sharply reduced in siRNA transfected groups in breast cancer cells (Fig. 5). Iturin A treatment caused significant Akt inhibition in non transfected cells as well as pcDNA-Akt transfected cells. Further, Iturin A caused enhanced inhibition of Akt in siRNA transfected groups. Similar kinds of results were also found in P-MAPK expression (Fig. 5).

**Figure 6:**
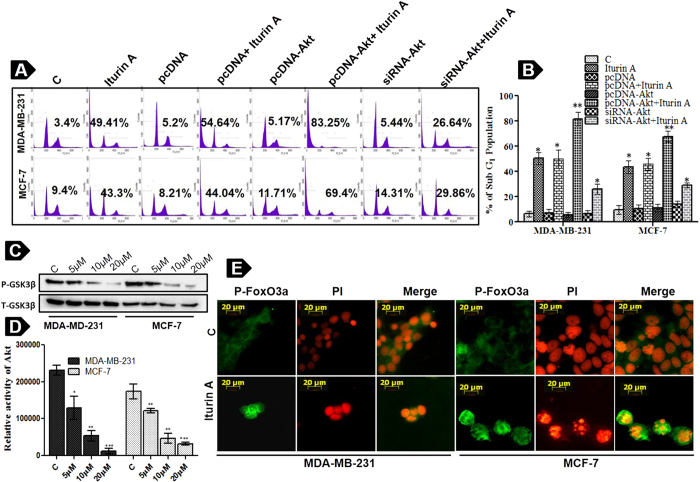
Cell cycle analysis of transfected cells, *in vitro* Akt kinase assay and translocation study of FoxO3a. (**A**) Flow cytometric analysis of transfected cells is performed to measure Sub G_1_ population. Transfected cells are treated with IC_50_ dose of Iturin A for 48 h and subjected to cell cycle analysis after staining with propidium iodide. (**B**) Graphical representations of Sub G_1_ population from flow cytometric analysis. (**C**) *In vitro* Akt kinase assay is performed using GSK3 fusion protein according to manufacture protocol. Blots were cut and cropped according to the molecular weight of the proteins. (**D**) Densitometric analysis of P-GSK3β level from kinase assay. Each bar represents mean ± S.D from three different experiments. Significance level is represented by *P<0.05, **P<0.01, ***P<0.001. (E) Breast cancer cells are treated by Iturin A with respective IC_50_ doses for 48 h. Florescent images are taken after incubation with primary antibody followed by FITC tagged secondary antibody. P-FoxO3a is found mostly in cytoplasm in EGF treated cancer cells. Iturin A treatment causes nuclear accumulation of P-FoxO3a in both cell lines. Florescent images are taken at 20 × magnification.

Quantitative apoptotic effect of Iturin A was determined by flow cytometry in different transfected and non-transfacted groups (Fig. 6A, B). Non transfected and pcDNA (empty vector) transfected groups displayed 49.41% and 54.64% Sub G_1_ fractions in Iturin A treated MDA-MB-231cells (Fig. 6A). Iturin A treated MCF-7 cells also showed 43.3% and 44.04% Sub G_1_ fractions in non transfected and pcDNA transfected cells respectively (Fig. 6A). Interestingly, Iturin A treated pcDNA-Akt transfected groups exhibited enhanced Sub G_1_ fractions 83.25% and 69.4% in both cell lines (Fig. 6A). However, only 26.64% and 29.86% Sub G_1_ fractions were observed in siRNA-Akt transfected groups of treated MDA-MB-231 and MCF-7 cells.

### Iturin A inhibits Akt kinase activity in breast cancer cells

Akt kinase assay based on nonradioactive method offers the measurement of kinase activity in the cells. This assay was performed using protein extract of MDA-MB-231 and MCF-7 cells treated with Iturin A. Our results showed significant dose dependent downregulation of P-GSK3β expression in treated groups compared to controls. These results reflected the Akt inhibitory effect of Iturin A in both cell lines (Fig. 6C, D).

### Iturin A causes nuclear localization of FoxO3a in breast cancer cells

FoxO3a is a downstream protein of Akt signaling pathway. FoxO3a acts as tumor suppressor through inducing apoptosis and cell cycle arrest. Activation of Akt leads to shifting of FoxO3a from nucleus to cytoplasm as well as inhibition of transcriptional activity of it^19^. We investigated cellular localization of FoxO3a in MDA-MB-231 and MCF-7 cells in response to Iturin A treatment. It was observed that FoxO3a was mostly present in cytoplasm in EGF exposed groups. Translocation of FoxO3a to nucleus from cytoplasm was observed in Iturin A treated groups (Fig. 6E). These results suggested that Iturin A induced nuclear accumulation of FoxO3a to mediate apoptosis in MDA-MB-231 and MCF-7 cells.

### *In vivo* antitumor effect of Iturin A in MDA-MB-231 mouse xenografts model

**Figure 7:**
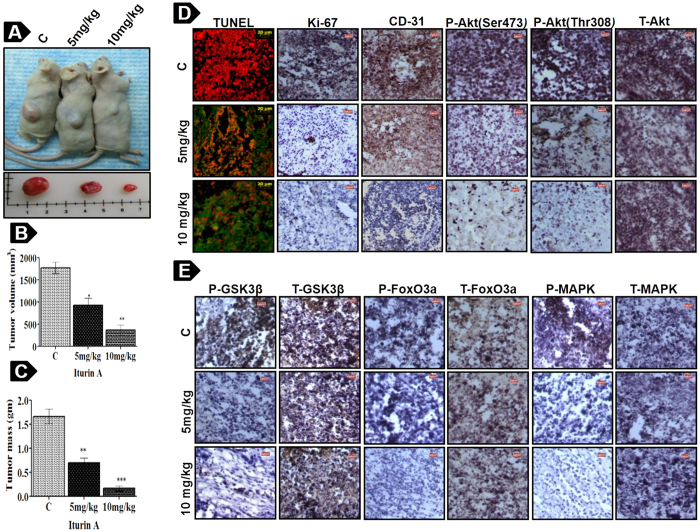
Anti-tumor activity of Iturin A in MDA-MB-231 human breast cancer xenografts. Nude mice bearing MDA-MB-231 xenografts treated with Iturin A (5 and 10 mg/kg). (**A**) Tumor bearing nude mice and excised tumors at the end of experiment. (**B**, **C**) Excised tumor volume (mm^3^) and mass (gm) are shown by representative bar graph. Significance level is represented by *P<0.05, **P<0.01, ***P<0.001. (**D**) TUNEL assay and immunohistochemistry of Ki-67, CD-31, P-Akt (Ser 473 and Thr 308) and T-Akt are performed from excised tumors tissue samples. (**E**) Immunohistochemitry of P-GSK3β, T-GSK3β, P-FoxO3a, T-FoxO3a, P-MAPK and T-MAPK.

Antitumor effect of Iturin A was tested on *in vivo* MDA-MB-231 xenograft model in nude mouse. After four weeks of treatment, there was significant reduction in tumor mass and tumor volume in treated groups compared to untreated controls (Fig. 7A, B, C). Visible toxicity in each mouse was not found during the period of study. Immunohistochemistry of tumor tissue sections showed increase in TUNEL positive cells and reduced expression of Ki-67 suggesting *in vivo* apoptotic and antiproliferative effect of Iturin A (Fig. 7D). Decrease phosphorylation of Akt (Ser473 and Thr308), GSK3β, FoxO3a and MAPK was also observed in treated groups by immunohistochemical analysis of tumor tissue sections (Fig. 7D, E). But expression of total proteins was unaltered.

## Discussion

Marine microorganisms currently represent a diverse and unexplored bioresources of valuable bioactive molecules. Among these bioactive molecules, lipopeptides show great promise in the development of the new chemotherapeutic agents. Previously, we have systematically reviewed the potential role of a number of lipopeptides e.g., surfactin, somacystinamide A, fengycin, pseudofactin, apratoxin and rakicidin as new generation anticancer agents^20^. In the current study, we isolated and purified Iturin A which is a lipopeptide molecule. This lipopeptide was reported to possess anti-fungal property against variety of fungi^21^. However, its anticancer property was not reported earlier. The aim of present study was to investigate *in vitro* and *in vivo* anticancer activity of Iturin A. Our study also demonstrated possible molecular mode of actions in Iturin A induced apoptosis in breast cancer.

Isoforms of Iturin A produced by marine *Bacillus megaterium* was purified by RP-HPLC and purity level of the isoforms was confirmed by comparing with that of the standard Iturin A from Sigma Aldrich, St. Louis, MO, USA. The isoform containing longer fatty acid chain was selected for further pharmacological studies as it effectively interacts with the cell membrane^22^.

The MTT assay is a colorimetric assay to determine cell cytotoxicity of anticancer molecules. This assay revealed dose dependent cytotoxic effects of Iturin A against different breast cancer cells MDA-MB-231, MCF-7, T47D and MDA-MB-468 (Fig. 2B). Iturin A showed more potent cytotoxic action on MDA-MB-231 and MCF-7 cells compared to other cell lines. Iturin A caused prominent morphological changes like membrane blebbing, loss of lamellipodia/filopodia and cellular shrinkage (Apoptotic features) in treated MDA-MB-231 and MCF-7 cells but no significant effects in normal HMEC and HaCaT cells (Fig. 2E). Further, Sub G_1_ accumulation of apoptotic cells was observed following treatment with Iturin A with different time points (Fig. 2C and D). Interestingly, human mammary epithelial cells HMEC and normal keratinocyte HaCat showed insignificant Sub G_1_ accumulation corroborating with morphological study of normal cells (Fig. 2C and D). All these data indicated selective cytotoxic and apoptotic effect of Iturin A on breast cancer cells.

DNA fragmentation is a hallmark of apoptosis caused by activation of endogenous endonucleases followed by cleavage of chromatin DNA. Prominent ladders formation indicated presence of fragmented DNA reflecting the involvement of apoptosis in Iturin A treated groups of MDA-MB-231 and MCF-7 cells (Fig. 3A).

Next, we performed western blot analysis to assess the profile of various pro and anti-apoptotic proteins as these proteins are regulated by Akt signaling^11^,^12^. Our finding showed downregulation of anti-apoptotic proteins Bcl-2, Bcl-xL and Mcl-1 as well as upregulation of apoptotic protein BAX in Iturin A treated MDA-MB-231 and MCF-7 cells (Fig. 3B, C, D). Our study described that elevated BAX to Bcl-2 ratio exerted apoptotic effect through mitochondrial Cytochrome C release, leading to activation of caspase3/7, PARP cleavage and DNA fragmentation. So, caspase dependent cell death was involved in apoptosis mediated by Iturin A on breast cancer cells.

It is now established that abnormal activation of Akt kinase is involved in many cancers including breast cancer^23^. So, targeting Akt kinase is a great promise for anticancer drug discovery. A number of compounds e.g. Perifosine, Erufosine, KP372-1, Triciribine, GSK690693 and MK2206 have been reported to target Akt signaling pathway for their anticancer actions^6^. Here, we investigated that whether Iturin A could inhibit Akt leading to apoptosis. Interestingly, current study revealed that Iturin A inhibited phosphorylation of Akt at both sites (Ser 473 and Thr308) in MDA-MB-231 and MCF-7 cells (Fig. 4). Next we assessed the inhibitory effect of Iturin A on Akt kinase activity by nonradioactive kinase assay kit in cell free system. This assay for *in vitro* measurement of Akt kinase activity showed diminished phosphorylation of fusion protein GSK in Iturin A treated breast cancer cells. This phenomenon reflected the inhibitory effect of Iturin A on Akt kinase (Fig. 6C, D). After that we hypothesized that Iturin A could inhibit phosphorylation state of Akt substrates FoxO3a and GSK3β to confirm Akt inhibition. FoxO3a is a downstream protein and forkhead family of transcription factors of Akt signaling pathway. Activated Akt phosphorylates FoxO3a and prevents apoptotic effects of FoxO3a by inhibiting nuclear accumulation. FoxO3a acts as tumor suppressor by inducing apoptosis and cell cycle arrest. So, Akt inhibition indirectly may cause reactivation and nuclear export of FoxO3a^24^. Our results confirmed that phosphorylation of FoxO3a was decreased after Iturin A treatment without altering total proteins (Fig. 4). Next, we assessed nuclear accumulation study of FoxO3a in Iturin A treated breast cancer cells. FoxO3a, when it is activated accumulates in nucleus and initiates transcriptional activity^25^. Our study demonstrated that Iturin A caused nuclear accumulation of FoxO3a in Iturin A treated MDA-MB-231 and MCF-7 cells. Iturin A suppressed Akt activity and indirectly inhibited phosphorylation of FoxO3a (Fig. 6E). Our results are similar to the previous report that reduction of FoxO3a phosphorylation level and nuclear localization were indirectly caused by paclitaxel mediated inhibition of Akt^26^. Another Akt substrate GSK3β is negatively modulated by Akt activity. Activated GSK3β (Non phosphorylated state) is reported to regulate cell cycle analysis and apoptosis^27^. Akt prevents the apoptotic activity of GSK3β by phosphorylating it. Our results suggested that Iturin A inhibited Akt activity and their by indirect inhibition of its substrate P-GSK3β (Fig. 4). In addition, transfection study provided the validation that Iturin A could suppress Akt kinase activity and block its signaling pathway *in vitro*. Increase activity of Akt was detected in pcDNA-Akt plasmid transfected groups and reduced expression of Akt was detected in siRNA-Akt transfected cells (Fig. 5). Iturin A treatment inhibited expression of Akt in both transfected and non-transfected groups of MDA-MB-231 and MCF-7 cells. Enhanced apoptotic effects were observed in Iturin A treated MDA-MB-231 and MCF-7 cells stably transfected with Akt plasmid. Interestingly, reduced apoptotic effects were observed in Iturin A treated breast cancer cells transfected with siRNA-Akt (Fig. 6A, B). These results are similar to the effect of another Akt inhibitor, triciribine which inhibits Akt over expressing cells more potently than those low Akt expressing cells^28^. So, apoptotic effects of Iturin A depend on Akt activity in cancer cells.

The present study also reported *in vivo* antitumor activity of Iturin A on nude mice transplanted with MDA-MB-231 cells. Tumor volume, mass and expression of various proteins were measured to assess antitumor property of Iturin A (Fig. 7A, B, C). Our study demonstrated markedly reduction of tumor volume and mass in a dose dependent fashion. TUNEL assay displayed presence of DNA fragmentation (TUNEL positive cells) in tumor tissue section of Iturin A treated groups (Fig. 7D). Immunohistochemical staining of tumor tissue indicated that Ki-67 and CD-31 expression were reduced in Iturin A treated groups compared to untreated controls. All these findings indicated *in vivo* antiproliferative and apoptotic potential of Iturin A in mouse xenografts model. Further, we investigated expression of Akt and its downstream proteins by immunohistochemical analysis. Reduced expression of P-Akt (Ser473 and Thr308) and its downstream substrates P-GSK3β and P-FoxO3a (Fig. 7D, E) was observed in Iturin A treated groups. Phosphorylation of MAPK was also inhibited in Iturin A treated groups (Fig. 7E).

In conclusion, the current study suggested that Iturin A of marine bacterial origin could inhibit Akt signaling network leading to apoptosis induction in breast cancer cells. This agent also inhibited tumor growth in breast cancer xenograft model. Our results also validated the discovery of Iturin A for therapy of breast cancer with elevated level of Akt. These results of the preclinical studies are very encouraging and promising for the clinical development of an Iturin A mediated Akt inhibitory mechanism based therapy for breast cancer in the near future.

## Materials and methods

### Chemicals and Reagents

For immunoblot and immunohistochemical analysis, various antibodies like rabbit monoclonal anti-p-MAPK, anti-MAPK, anti-p-Akt (Ser & Thr) and anti-Akt, anti-p-GSK-3β, anti-GSK-3β, anti-p-FoxO3a, anti-FoxO3a monoclonal mouse anti-CD-31, rabbit anti-caspase 3/7 were purchased from Cell Signaling Technology, Beverly, MA, USA. Mouse anti-Ki-67, rabbit monoclonal anti-PARP, mouse monoclonal anti-Bcl-2, anti-Bax, anti-Mcl-1, anti-Bcl-xL, HRP conjugated goat anti-rabbit IgG and goat anti-mouse IgG were purchased from Santa Cruz Biotechnology, Santa Cruz, CA, USA. Mouse monoclonal anti-β-actin and other reagents were procured from Sigma Aldrich, St. Louis, MO, USA. Antibodies dilutions have been made as per the manufacturer’s instruction. The pcDNA3-Akt-HA plasmid was obtained as gift sample from Dr. Guy Salvesen (University of California, San Diego, CA, USA). siRNA Akt and pcDNA3.1(-) were purchased from Cell Signaling Technology, Beverly, MA, USA and Invitrogen, USA respectively. Opti-MEM^®^I Reduced Serum Media and fetal bovine serum (FBS) were purchased from Gibco-BRL Invitrogen Corporation, CA, USA. FuGENE® HD transfection reagent was obtained from Roche Applied Science, Mannheim, Germany. Trypsin, Bovine serum albumin (BSA) and antibiotics (10,000 U/L penicillin and 10 mg/L streptomycin) were purchased from Himedia, Mumbai, India.

### Microorganism, media composition and culture conditions

A marine bacterium *Bacillus megaterium* was used for production of Lipopeptide^29^. This bacterial strain was obtained from sea water of the Andaman and Nicobar Islands, India. For preparation of the primary inoculums, bacterial strain was maintained in Zobell marine agar (Himedia, Mumbai, India) and grown in Zobell marine broth following incubation for 12 h at 32 °C. Primary inoculums were inoculated in 250 ml Erlenmeyer flask containing glucose mineral salts medium (GMSM containing: glucose−25 g/L, NH_4_NO_3_–6 g/L, KH_2_PO_4_–0.028 g/L, K_2_HPO_4_–1.6 g/L, MgSO_4_•7H_2_O–0.3 g/L and CaCl_2_•2H_2_O–0.2 g/L) and the flask was kept in a shaking incubator at 180 rpm and 32 °C till mid-exponential growth of cells. The inoculum was further inoculated in 1 L Erlenmeyer flasks containing 200 ml GMSM and kept in incubator at 180 rpm and 32 °C. Fermentation broth samples were collected at different time for lipopeptide collection.

### Cell lines and culture condition

Human breast cancer cell lines MDA-MB-231, MDA-MB-468, MCF-7, T47D and keratinocytes HaCaT were purchased from National Centre for Cell Science (NCCS), Pune, India. Human mammary epithelial cells (HMEC) were purchased from Lonza Clonetics, San Diego, USA and cultured in recommended medium. Other cells were grown in Dulbecco’s modification of Eagle’s medium (DMEM) supplemented with 3.7 g Sodium bicarbonate (Invitrogen Corporation, CA), antibiotics (10,000 U/L penicillin and 10 mg/L streptomycin) (Himedia, Mumbai, India) and 10% FBS. Adherent monolayer cell lines were maintained in plastic culture flask and incubated at 37 °C in 5% CO_2_ in humidified incubator.

### Isolation of marine bacterial lipopeptide

Iturin A produced by *Bacillus megaterium* was isolated according to earlier method^30^. After 28 h of cultivation, the cells were separated by centrifugation. Concentrated HCl was added in the culture broth to reduce the pH (up to 2.0).The precipitate was taken by centrifugation and extracted with methanol. Further analysis was performed with concentrated methanolic extracts.

### Purification and characterization of lipopeptide

The extract was purified using reverse phase HPLC instrument (Agilent technologies, CA, USA) equipped with Zorbax C_18_ column and photodiode array detector. The mobile phase was consisted of solvent A (MilliQ water) and solvent B (HPLC grade acetonitrile with 0.1% trifluoroacetic acid). The lipopeptides were eluted by linearly increasing the percentage of solvent B from 5–95% for 60 min at a flow rate of 0.4 ml/min. About 50 μl of methanol extracted sample and standard Iturin (Sigma, USA) was injected into the column. The HPLC fractions were collected automatically and subjected to mass spectrometry. Electrospray ionization mass spectrometry (ESI-MS) analysis was performed on EVOQ triple quadrupole mass spectrometer (Bruker). ESI-MS conditions were as follows: capillary voltage of 35 V, a spray voltage of 4.5 kV and a capillary temperature of 300 °C. Samples were injected with a syringe at a flow rate of 0.2 ml/min.

### Cell cytotoxicity assay of Iturin A

Iturin A fraction was collected from HPLC analysis, dried and dissolved in milliQ water (pH adjusted to 7.4) and stored at −20 °C. The stock solution was diluted in incomplete medium just before use. Cell cytotoxicity assay was performed by using MTT dye according to previous method^31^. MDA-MB-231, MDA-MB-468, MCF-7 and T47D cells were seeded in 96 well plate (2.5 × 10^3^ cells/well) and kept in incubator for 24 h to attach cells. After that, breast cancer cells were treated with different concentrations of Iturin A. Following 48 h of treatment, 100 μl of MTT solution (1 mg/ml in DMEM) was added in each well and the plates ware incubated for another 4 h at 37 °C. MTT dye is converted to purple formazan crystal due to mitochondrial reductase activity of live cells. The formazan crystal was dissolved in 100 μl Dimethyl sulfoxide (DMSO) per well and optical density was measured by microplate reader (Bio-Rad, Hercules, CA, USA) at 540 nm. Due to higher sensitivity, MDA-MB-231 and MCF-7 cells were chosen for further study.

### Flow cytometry based apoptosis assay

Apoptotic effects of Iturin A on the MDA-MB-231, MCF-7, HMEC and HaCaT cells were determined using flow cytometry analysis by previous method^32^. Briefly, 2.0 × 10^4^ cells/well were seeded in a 6-well plate and incubated for 24 h for cell growth. Incomplete medium was added in 6-well plate and it was kept in incubator overnight for starvation following time dependent (12, 24 and 48 h) treatment of Iturin A. After treatment, cells were collected by trypsinization and fixed with chilled ethanol. Following overnight fixation, cells were centrifuged and washed with PBS followed by propidium iodide staining. The cells were analyzed by flow cytometry.

### Cytoskeletal analysis by fluorescence microscopy

MDA-MB-231, MCF-7, HMEC and HaCaT cells were seeded on sterile lysine coated cover slips. Cells were treated with Iturin A for 12, 24 and 48 h. Adherent monolayer cells were washed with PBS and fixed with 4% paraformaldehyde. Cells were washed with PBS and permeabilized with 0.1% triton X-100 for 1 min. Rhodamine phalloidin staining was performed to visualize cell cytoskeleton. DAPI was used to stain nucleus. After staining, the adhering cells were washed three times with PBS, dried and mounted on slides. Fluorescent images were captured under a Zeiss Observer Z1 microscope (Carl Zeiss, Germany) at 20 × magnification^33^.

### DNA ladder assay

MDA-MB-231 and MCF-7 cells were treated with Iturin A for 12, 24 and 48 h along with control cultures in serum free medium^34^. Both suspended and adherent cells were collected and washed with PBS. Then cells were lysed by lysis buffer. After that, total DNA was isolated by DNA isolation kit (Sigma Aldrich, St. Louis, MO, USA). DNA fragmentation was checked by agarose gel electrophoresis.

### Western Blot Analysis

MDA-MB-231 and MCF-7 cells were seeded in 6-well plates with subsequent treatment with IC_50_ dose of Iturin A for 12, 24 and 48 h to check the expression of proteins involved in apoptosis. To evaluate expression of phospho proteins, cells were treated with Iturin A and stimulated by human recombinant protein EGF (25 ng/ml) for 30 min. Both suspended cells as well as adherent cells were collected and washed with PBS twice. Cells were lysed with NP-40 lysis buffer (Invitrogen Corporation, CA, USA). After that, western blot analysis was performed according to previous reported methods^35^.

### Akt kinase assay

The activity of Akt kinase was estimated by using Akt Kinase assay kit (Cell Signaling Technology, Beverly, MA, USA) according to manufacturer’s protocols. MDA-MB-231 and MCF-7 cells were treated with different doses of Iturin A for 12 h and Akt kinase activity was measured according to earlier reported method^36^.

### Transfection study

MDA-MB-231 and MCF-7 cells were plated in 70 mm Petri dishes in complete medium. Cells were starved for overnight with serum free medium. Cells were transiently transfected with 5 μg of pcDNA-Akt and empty vector with 7.5 μl of FuGENE® HD transfection reagent in 100 μl of Opti-MEM®I reduced serum media according to manufacturer’s protocol. After 24 h of transfection, the cells were treated with Iturin A for 24 h in serum free medium. siRNA-Akt was used to silence Akt expression. Briefly, cells were seeded in 70 mm Petri plates. After 70% confluency, cells were transfected with 14 mM siRNA-Akt in 6 μl DharmaFECT 4 Transfection Reagent (Thermo scientific) and 1 ml Opti-MEM^®^I reduced serum media according to manufacturer’s protocol. After 24 h of transfection, cells were treated with Iturin A for 24 h in serum free medium. After Iturin A treatment, both pcDNA-Akt and siRNA-Akt transfected cells were lysed with NP-40 lysis buffer and the protein expression profiles were checked by immunoblotting as discussed before. ImageMaster 2D Platinum 7.0 Software (GE Healthcare Life Sciences, NJ, USA) was used to plot comparative densitometric graph of blots. Iturin A treated transfected as well as control cells were also analyzed by flow cytometry for detection of apoptosis as per above described method^35^.

### Immunofluorescence staining for FoxO3a localization in breast cancer cells

To study nuclear accumulation of FoxO3a protein, indirect immunoflorescence assay was performed. Briefly, cells were grown on sterile lysine coated cover slips following treatment with Iturin A or EGF. Cell fixation was performed using 4% paraformaldehyde for 30 min and cells were permeabilized with 0.1% Triton X-100 solution for one minute. Cover slips were blocked with 5% BSA for one hour to remove non-specific bindings. Samples were incubated in primary antibody with appropriate dilution for overnight at 4 °C. Samples were incubated for 1 h in dark with FITC (Fluorescein isothiocyanate) tagged secondary antibody (Dako) after three times washing with PBST. Propidium iodide was used for nuclear staining. After drying, cover slips were mounted with D.P.X. and fluorescent images were captured under a Zeiss Observer Z1 microscope at 20 × magnification^37^.

### Inhibition of tumor in human breast cancer xenograft mouse model

*In vivo* antitumor activity of Iturin A was studied using human breast cancer nude mouse xenograft model. This study was approved by ministry of earth science (MOES), India; under the project number MOES/16/48/09/RDEAS. Animal experiment was carried out in accordance with the approved guidelines of Indian Council of Medical Research (ICMR), New Delhi, India. All experiment protocols were approved by institutional animal ethical committee of Indian Institute of Technology Kharagpur, India. Mice were maintained in aseptic condition at institute animal facility for seven days before injection with MDA-MB-231 cells. MDA-MB-231 cells (2 × 10^6^ cells in matrigel) were injected subcutaneously in 6–7 week old female athymic BALB/c (nu+/nu+) mice. Tumors were developed after 14 days and animals were randomly divided in three groups (Each group contains five animals) followed by measurement of tumor volume and body weight. Iturin A was injected by tail vein as following manner: (I) group1-control, (II) group2-Iturin A 5 mg/kg and (III) group 3-Iturin A 10 mg/kg. Animals were treated for four weeks in alternative days. Animals were observed regularly for tumor growth, survival, visible toxicity and changes in body weight during the study. After treatment, all animals were sacrificed to measure tumor volume and tumor weight. Tumor sections were fixed in formalin for immunohistochemical analysis^38^.

### TUNEL and immunohistochemistry of tissue sections

The apoptotic effect of Iturin A in mice tumor was evaluated by using commercial TUNEL kit (Roche) according to manufacturer’s protocols. Propidium iodide was used as counter stain. Immunohistochemistry for a number of proteins (Ki-67, P-Akt, T-Akt, P-GSK3β, T-GSK3β, P-MAPK and T-MAPK) was also performed by previous methods^31^.

### Software and Statistical analysis

GraphPad prism software was used to perform statistical analysis. Each experiment was carried out at least three times. The results with p-value<0.05 were considered significant. Image Master 2D Platinum 7.0 Software (GE Healthcare Life Sciences, NJ, USA) was used for densitometry analysis of blot. All data were represented as mean ± standard deviation (SD).

## Author Contributions

Design of the experiments: GD, RB, RKS, MM. Execution of the experiments: GD, RB, GDH. Analysis of the data: GD, RB, RKS, MM. Contribution in reagents/materials/analysis tools: GD, SD, KKD, BNP. Writing the paper: GD, RB, RKS, MM.

## Additional Information

**How to cite this article**: Dey, G. *et al*. Marine lipopeptide Iturin A inhibits Akt mediated GSK3ß and FoxO3a signaling and triggers apoptosis in breast cancer. *Sci. Rep.* **5**, 10316; doi: 10.1038/srep10316 (2015).
